# The 2^nd^ sialic acid-binding site of influenza A virus neuraminidase is an important determinant of the hemagglutinin-neuraminidase-receptor balance

**DOI:** 10.1371/journal.ppat.1007860

**Published:** 2019-06-10

**Authors:** Wenjuan Du, Hongbo Guo, Vera S. Nijman, Jennifer Doedt, Erhard van der Vries, Joline van der Lee, Zeshi Li, Geert-Jan Boons, Frank J. M. van Kuppeveld, Erik de Vries, Mikhail Matrosovich, Cornelis A. M. de Haan

**Affiliations:** 1 Virology Division, Faculty of Veterinary Medicine, Utrecht University, Utrecht, The Netherlands; 2 Institute of Virology, Philipps University, Marburg, Germany; 3 Department of Chemical Biology and Drug Discovery, Utrecht University, Utrecht, the Netherlands; Icahn School of Medicine at Mount Sinai, UNITED STATES

## Abstract

Influenza A virus (IAV) neuraminidase (NA) receptor-destroying activity and hemagglutinin (HA) receptor-binding affinity need to be balanced with the host receptor repertoire for optimal viral fitness. NAs of avian, but not human viruses, contain a functional 2^nd^ sialic acid (SIA)-binding site (2SBS) adjacent to the catalytic site, which contributes to sialidase activity against multivalent substrates. The receptor-binding specificity and potentially crucial contribution of the 2SBS to the HA-NA balance of virus particles is, however, poorly characterized. Here, we elucidated the receptor-binding specificity of the 2SBS of N2 NA and established an important role for this site in the virion HA-NA-receptor balance. NAs of H2N2/1957 pandemic virus with or without a functional 2SBS and viruses containing this NA were analysed. Avian-like N2, with a restored 2SBS due to an amino acid substitution at position 367, was more active than human N2 on multivalent substrates containing α2,3-linked SIAs, corresponding with the pronounced binding-specificity of avian-like N2 for these receptors. When introduced into human viruses, avian-like N2 gave rise to altered plaque morphology and decreased replication compared to human N2. An opposite replication phenotype was observed when N2 was combined with avian-like HA. Specific bio-layer interferometry assays revealed a clear effect of the 2SBS on the dynamic interaction of virus particles with receptors. The absence or presence of a functional 2SBS affected virion-receptor binding and receptor cleavage required for particle movement on a receptor-coated surface and subsequent NA-dependent self-elution. The contribution of the 2SBS to virus-receptor interactions depended on the receptor-binding properties of HA and the identity of the receptors used. We conclude that the 2SBS is an important and underappreciated determinant of the HA-NA-receptor balance. The rapid loss of a functional 2SBS in pandemic viruses may have served to balance the novel host receptor-repertoire and altered receptor-binding properties of the corresponding HA protein.

## Author summary

Influenza A viruses infect birds and mammals. They contain receptor-binding (HA) and receptor-destroying (NA) proteins, which are crucial determinants of host tropism and pathogenesis. It is generally accepted that the functional properties of HA and NA need to be well balanced to enable virion penetration of the receptor-rich mucus layer, binding to host cells, and release of newly assembled particles. This HA-NA-receptor balance is, however, poorly characterized resulting in part from a lack of suitable assays to measure this balance. In addition, NA is much less studied than HA. NA contains, besides its receptor-cleavage site, a 2^nd^ receptor-binding site, which is functional in avian, but not in human viruses. We now show that this 2^nd^ receptor-binding site prefers binding to avian-type receptors and promotes cleavage of substrates carrying this receptor. Furthermore, by using novel assays, we established an important role for this site in the HA-NA-receptor balance of virus particles as it contributes to receptor binding and cleavage by virions, the latter of which is required for virion movement and self-elution from receptors. The results may provide an explanation for the rapid loss of a functional 2^nd^ receptor-binding site in human pandemic viruses.

## Introduction

Influenza A virus (IAV) particles contain hemagglutinin (HA) and neuraminidase (NA) glycoproteins. HA functions as a sialic acid (SIA)-binding and fusion protein. NA has receptor-destroying activity by cleaving SIAs from sialoglycans. The HA and NA protein functionalities are critical for host tropism, and need to be well balanced in relation to the host receptor repertoire for optimal in vivo viral fitness [1–3]. However, there is no standard assay and unit for measuring a functional balance and the precise mode by which HA- and NA-receptor interactions contribute to the balance at the molecular level remains mostly unexplored. An optimal HA-NA balance is hypothesized to allow virions to penetrate the heavily sialylated mucus layer, to attach to host cells prior to virus entry, and to be released from cells after assembly [4–7].

Aquatic birds constitute the natural reservoir of IAVs. Occasionally IAVs from birds cross the host species barrier and manage to adapt to non-avian species, including humans. The human receptor repertoire differs from avians and requires adaptations in the SIA-interacting HA and NA proteins for optimal interaction. The HA protein of avian IAVs prefers binding to terminally located SIAs linked to the penultimate galactose via an α2,3-linkage. Human IAVs preferentially bind to α2,6-linked sialosides [8–11]. Internal sugars and their linkages as well as glycan branching have been shown to determine fine specificity of HA-receptor binding [12–17]. Changes in the receptor-binding properties of the HA proteins are achieved by mutations in the receptor binding site, which have been well documented for several HA subtypes [1, 10, 11, 18]. Much less is known about the adaptations in NA required to match the corresponding HA proteins.

NA is a type II transmembrane protein that forms mushroom-shaped homotetramers. Tetramerization is essential for its enzymatic activity [19, 20]. The enzyme active site is located in the globular head domain that is linked to the endodomain via a thin stalk. The active site is made up by catalytic residues that directly contact SIA and by framework residues that keep the active site in place [21, 22]. The catalytic and the framework residues are extremely conserved between avian and human IAVs [23]. Nevertheless, although both avian and human NA proteins preferentially cleave α2,3-linked SIAs, human viruses appear relatively better at cleaving α2,6-linked SIAs [24–27].

Adjacent to the catalytic site, NA contains a 2^nd^ SIA-binding site (2SBS; also referred to as hemadsorption site) (S1 Fig)[28–31]. The 2SBS is made up by three loops, which contain residues that interact with SIA. Mutations in these loops in N1, N2 and N9 affected NA binding of erythrocytes [28, 32–35] or sialosides [26, 33] and enzymatic cleavage of multivalent substrates [28, 33] but not of monovalent substrates [26, 28, 33]. A detailed analysis of the receptor binding properties of the 2SBS of most NAs is lacking. N1 and N2 proteins bind to α2,3- as well as α2,6-linked SIAs based on binding of resialylated erythrocytes [28, 35] whereas N1 and N9 proteins mainly bind, via their 2SBS, to α2,3-linked sialosides present on glycan arrays [33] or in biolayer interferometry assays [26]. Interestingly, the high conservation of SIA-contact residues in the 2SBS of avian IAV is lost in N1 and N2 of human IAVs [1, 26, 28, 30] that, supposedly, all lack a functional 2SBS. For N2 of avian viruses, the conservation of the 2SBS is only lost in viruses of the H9N2 subtype, which mainly infect *Galliformes* species, in contrast to other N2-containing viruses, which mainly infect Non-*Galliformes* species [36] (S2 Fig). Conservation of the SIA-contact residues in the 2SBS of N2 is also lost in canine and not restored in swine viruses, the latter of which are generally derived from human viruses (S2 Fig). It is tempting to hypothesize that the loss of a functional 2SBS in pandemic viruses is part of a required adaptation of the HA-NA balance in order to deal with the altered receptor repertoire in the novel human host [1, 28]. At first, to test this hypothesis, a detailed analysis of the contribution of (mutations in) the 2SBS to receptor binding and cleavage in the context of IAV particles is necessary as the interplay with HA proteins binding to either avian- or human-type receptors needs to be taken into account.

We define the HA-NA balance as the balance between the activities of HA and NA in virus particles in relation to their functional receptors on cells and decoy receptors present e.g. in mucus. We have recently established novel kinetic assays based on biolayer interferometry (BLI) with which, in the context of virus particles, HA binding, NA cleavage and their balance can be monitored in real time using synthetic glycans and sialylated glycoproteins [37]. Multivalent IAV-receptor binding is established by multiple low affinity interactions of several HA trimers and sialosides [38, 39]. This enables a dynamic binding mode in which individual interactions are rapidly formed and broken without causing dissociation of the virus but providing access of NA to temporarily free SIAs. Cleavage by NA results in reduced SIA-receptor density, in virus movement and ultimately in virion dissociation [37]. How fast this occurs depends on the HA-NA-receptor balance governing the dynamics of virus-glycan interactions.

In the present study, we applied these novel BLI assays to study the HA-NA-receptor balance of viruses that have a single amino acid substitution in the 2SBS. We first performed a detailed analysis of the functional importance of the 2SBS in N2 for substrate binding and cleavage by comparing NA of the pandemic H2N2 virus from 1957, containing a mutated 2^nd^ SIA-binding site, with an avian-like NA, in which the 2^nd^ SIA binding site was restored. Preferred binding to α2,3-linked sialosides was shown to result in enhanced cleavage of substrates containing these glycans. Analysis of the HA-NA-receptor balance of viruses containing these N2 proteins in combination with H3 proteins that prefer binding to avian or human-type receptors clearly demonstrated a role for the 2SBS in the complex and dynamic interplay between HA, NA and receptor, which has been largely overlooked until now. The functional importance of the 2SBS for the HA-NA-receptor balance may explain the conservation and loss of this site in avian and human IAVs, respectively.

## Results

### The 2SBS in N2 is an important determinant of NA catalytic activity

**Fig 1:**
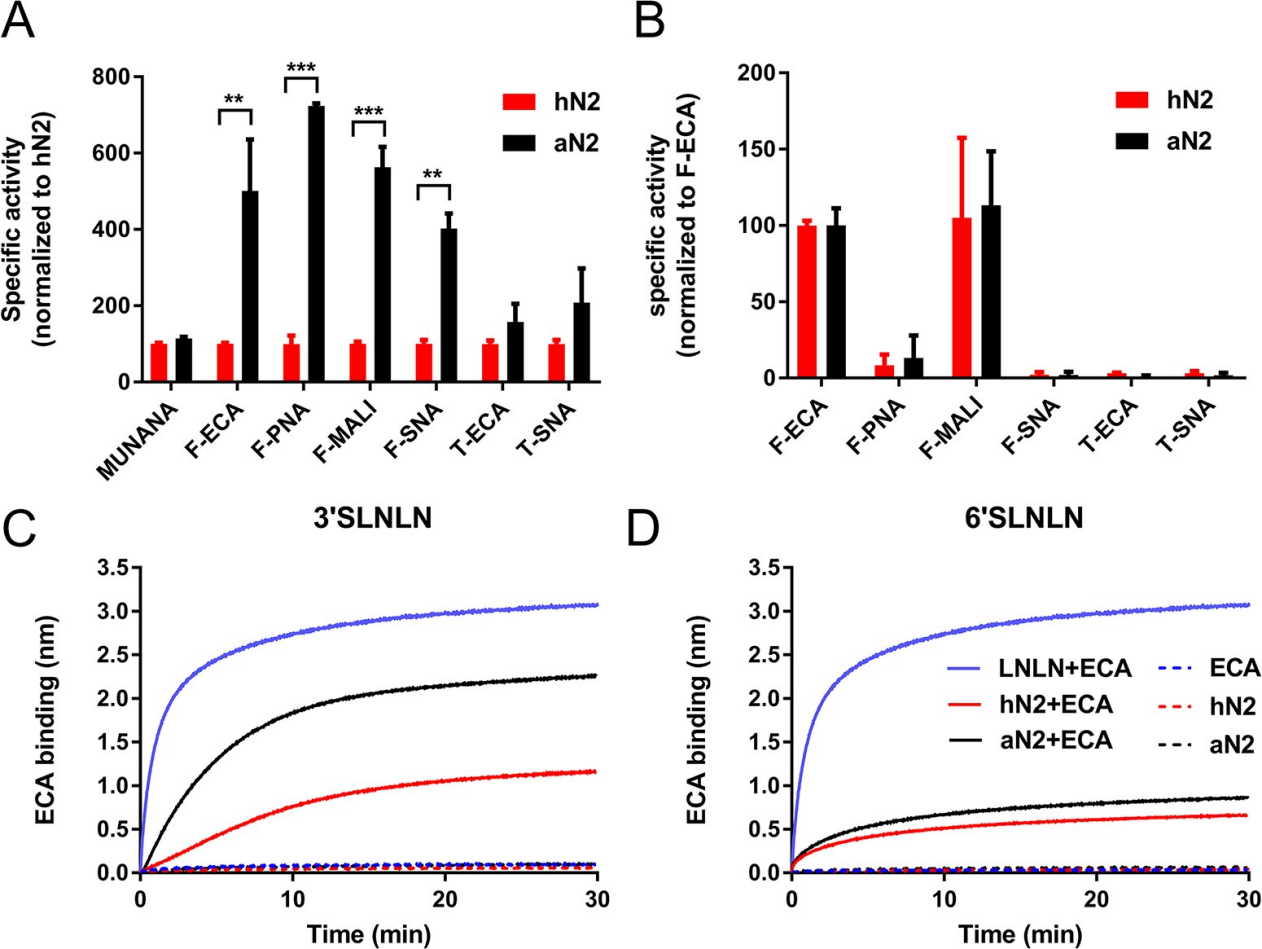
Enzymatic activity of N2 proteins assayed using different substrates. (A) Specific activity of hN2 and aN2 was determined by MUNANA assay and ELLA using different glycoprotein-lectin combinations (Fetuin-ECA, Fetuin-PNA, Fetuin-MAL I, Fetuin-SNA, Transferrin-ECA and Transferrin-SNA) and normalized to the specific activity of hN2 for MUNANA and each glycoprotein-lectin combination. (B) Specific activity of hN2 and aN2 is graphed normalized to the specific activity as determined for each protein by the Fetuin-ECA combination. Mean values and standard deviations from two independent experiments performed in triplicate are shown. Stars depict P values calculated using an unpaired two-tailed Student t test (**, P<0.01; ***, P<0.001). (C and D) BLI kinetic assay of NA enzymatic activity. Streptavidin biosensors were coated with biotinylated synthetic glycans (3’SLNLN, 6’SLNLN or LNLN). Subsequently, the sensors were incubated in buffer containing 4 μg aN2 or hN2 in the absence or presence of 8 μg ECA or ECA alone. ECA binding to sensors coated with 3’SLNLN or 6’SLNLN is a measure for SIA cleavage from these receptors by NA. Experiments were independently performed three times. Representative experiments are shown.

We first analysed the receptor binding and cleavage activity of N2 NA with and without a functional 2SBS using purified recombinant soluble NA expressed in HEK293T cells [20]. In NA of A/Singapore/1/1957 (H2N2) pandemic virus (referred to as human N2 [hN2]) one of the SIA-contact residues in the 2SBS is mutated compared to the avian consensus sequence (S367N, S3 Fig). Introduction of the reciprocal mutation (N367S) in this NA restored the 2SBS (referred to as avian-like N2 [aN2]) [28]. hN2 and aN2 displayed similar specific activities when using the monovalent MUNANA [2’-(4-Methylumbelliferyl)-α-D-N-acetylneuraminic acid] substrate (Fig 1A, S4A and S4B Fig), indicating that mutation of the 2SBS did not affect the catalytic activity of the N2 proteins *per se*. Similar results were obtained previously using membrane-associated proteins [28], indicating that the activity of the recombinant soluble proteins accurately reflects the activity of their membrane-bound counterparts as concluded earlier for N1 [20]. Cleavage of SIAs from fetuin and transferrin sialoglycoproteins was quantified by enzyme-linked lectin assay (ELLA), by analysing the increase or decrease in binding of lectins depending on their binding specificities (S4 Fig). ECA (*Erythrina Cristagalli* lectin) specifically binds glycans containing terminal Galα1,4GlcNAc corresponding to non-sialylated N-linked sugars [40], while PNA (peanut agglutinin) binds to terminal Galβ1,3GalNAc, which generally corresponds to non-sialyated O-linked sugars [41]. NA activity thus results in increased binding of these lectins. MAL I (*Maackia Amurensis* Lectin I) and SNA (*Sambucus Nigra* Lectin) specifically bind α2,3- or α2,6-linked SIAs, respectively [42, 43]. Binding of SNA and MAL I is decreased by NA activity. For all lectins analysed, aN2 was more active than hN2 using fetuin, containing α2,3- and α2,6-linked SIAs (Fig 1A) [44]. In contrast, no statistically significant difference was observed using transferrin that only contains α2,6-linked sialoglycans (Fig 1A) [45, 46]. Plotting the specific activities of the NA proteins relative to their specific activities as determined by the fetuin-ECA combination resulted in similar activity profiles (Fig 1B), which mimic those determined previously for N1 and N9 [26, 33]. Both hN2 and aN2 preferred cleavage of α2,3- (determined with fetuin-MAL I) over α2,6-(determined with fetuin-SNA) linked SIAs (Fig 1B). In agreement herewith, the specific activities were higher when determined with the fetuin-ECA than with the transferrin-ECA combination as fetuin, but not transferrin, contains α2,3-linked SIAs (Fig 1B).

These results show that an avian-like 2SBS in N2 contributes to cleavage of the sialoglycoprotein fetuin containing α2,3- and α2,6-linked SIAs. We next used BLI to study the kinetics of NA activity on a multivalent surface coated with either an avian receptor (3’SLNLN: NeuAcα2-3Galβ1-4GlcNAcβ1-3Galβ1-4GlcNAc) or a human receptor (6’SLNLN: NeuAcα2-6Galβ1-4GlcNAcβ1-3Galβ1-4GlcNAc). NA activity can be directly monitored in real-time by the specific binding of the lectin ECA to terminal Galβ1-4GlcNAc glycotopes that become available upon removal of SIA by NA (Fig 1C and 1D, red and black lines). Note that cleavage of the small SIA moiety is not detected directly by BLI (Fig 1C and 1D, dashed red and black lines). Binding of ECA to a sensor coated with LNLN (Galβ1-4GlcNAcβ1-3Galβ1-4GlcNAc, Fig 1C and 1D blue lines) rapidly reaches the maximum ECA binding signal (representing 100% de-sialylation) assuring that ECA binding during the relatively slow accumulation of de-sialylated glycans by NA activity (red and black lines) reflects the cleavage kinetics of the N2 proteins. Both hN2 and aN2 more efficiently cleaved 3’SLNLN over 6’SLNLN. Especially the aN2 protein displayed much more efficient cleavage of 3’SLNLN. We conclude that restoration of the 2SBS in hN2 to the avian consensus sequence results in enhanced cleavage of substrates containing α2,3-linked SIAs.

### N2 proteins prefer binding to α2,3- over α2,6-linked SIAs via their 2SBS

**Fig 2:**
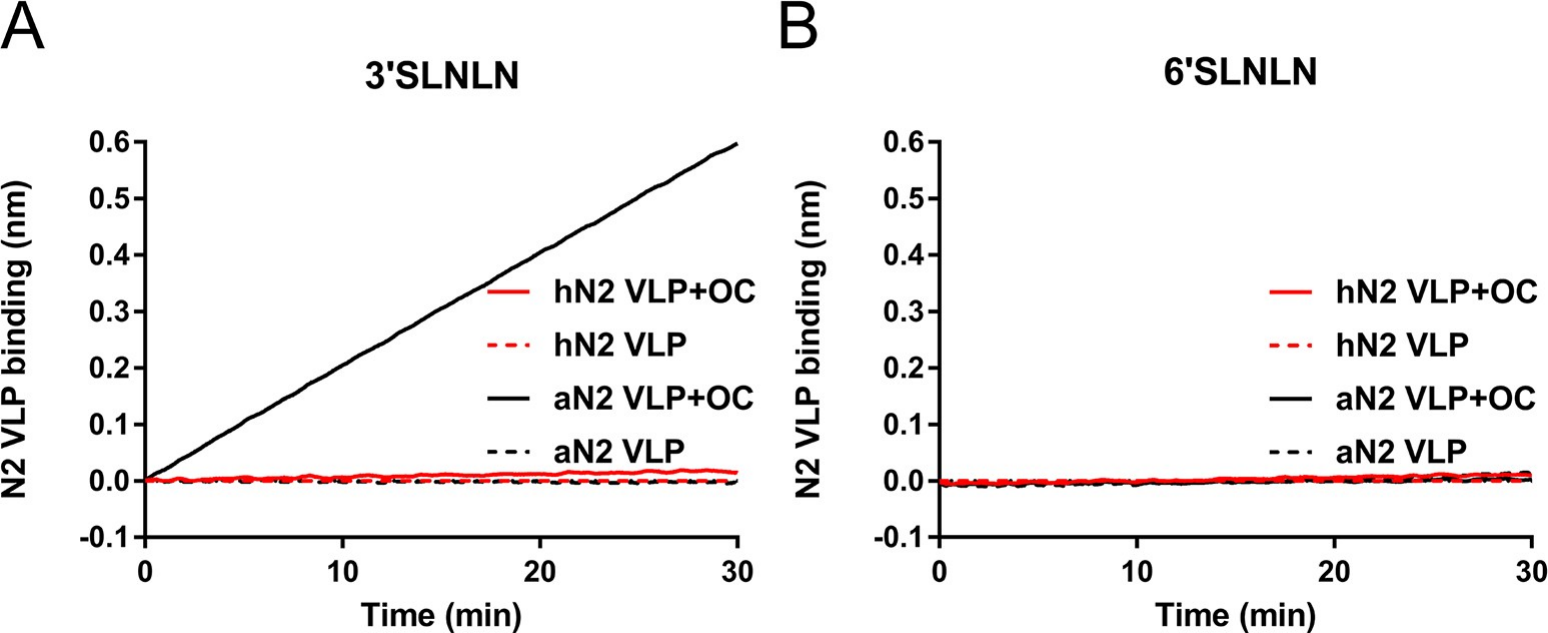
Substrate binding of N2 VLPs via the 2SBS. VLPs containing full length hN2 and aN2 were analysed for their ability to bind 3’SLNLN (A) or 6’SLNLN (B) in the absence or presence of OC using BLI as described in the legend to Fig 1. Similar amounts of N2 were applied based on the MUNANA assay as hN2 and aN2 proteins have identical enzymatic activities with respect to this substrate (Fig 1). Experiments were independently performed twice. Representative experiments are shown.

The increased cleavage by aN2 of substrates containing α2,3-linked SIAs is expected to result from specifically increased binding to α2,3-linked SIAs due to the presence of an avian 2SBS, although N2 proteins were reported to bind both α2,3- and α2,6-linked SIAs by using resialylated erythrocytes [28]. We observed hemagglutination for recombinant soluble aN2 but not for hN2 (S5A Fig). However, no specific binding to synthetic α2,3- and α2,6-linked sialoglycans by BLI could be observed for the recombinant soluble N2 proteins, which could be due to low affinity of the 2SBS. By embedding the N2 proteins in membrane vesicles highly multivalent receptor interactions may increase receptor-binding avidity. To this end, full length N2 proteins were expressed in 293T cells. N2 virus-like particles (VLPs) [47] were directly harvested from the culture supernatant, while cells were treated with hypotonic and hypertonic buffers, resulting in the release of N2 protein-containing vesicles [48]. Preparations containing similar amounts of N2, based on MUNANA activity (Fig 1A) were used to determine the receptor specificity of the 2SBS by BLI [26]. Negligible binding was obtained for hN2 VLPs (Fig 2A and 2B) or vesicles (S5D and S5E Fig) to α2,3- or α2,6-linked SIAs, regardless of the presence of the NA inhibitor oseltamivir carboxylate (OC), which binds the NA catalytic site. In contrast, highly 3’SLNLN-specific, binding was observed for aN2 VLPs and vesicles (Fig 2A and 2B; S5D and S5E Fig) in the presence of OC leading to the conclusion that aN2 has much higher lectin activity than hN2 due to the presence of a functional 2SBS. The observed α2,3-linked SIA specificity is in agreement with the particularly enhanced cleavage of substrates containing α2,3-linked SIAs (Fig 1). No binding of aN2 VLPs to 3’SLNLN was observed in the absence of OC, which is likely explained by immediate self-elution of VLPs carrying active NA proteins.

### Restoration of the N2 2SBS affects virus replication

**Fig 3:**
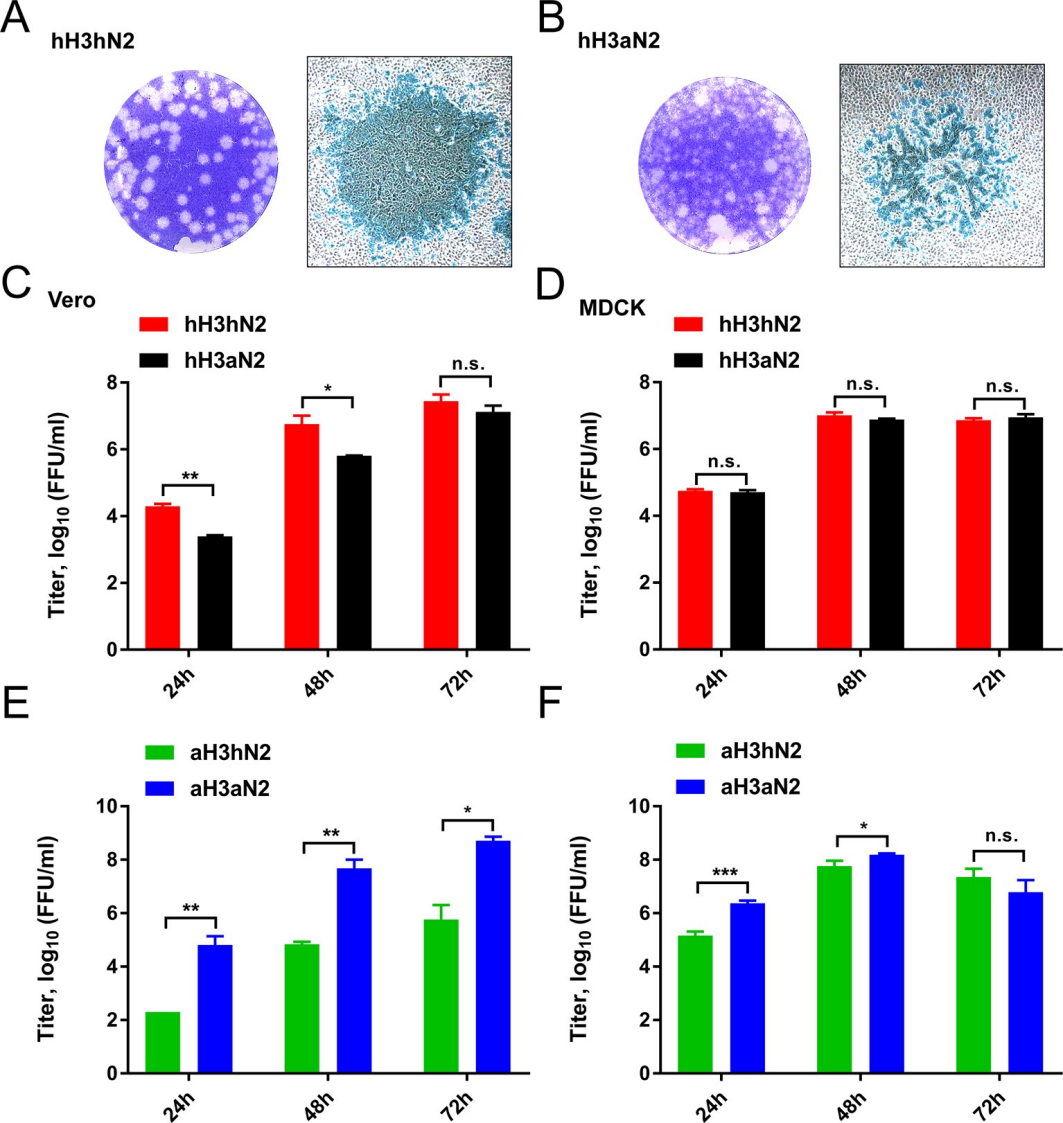
Plaque morphology and replication kinetics of recombinant viruses. Plaque assays were performed for hH3hN2 (A) and hH3aN2 (B) viruses using Vero cells followed by crystal violet dye staining (left panels) or by immunostaining of infected cells (right panels). Vero (C and E) or MDCK (D and F) cells were infected with hH3hN2 or hH3aN2 (C and D), or with aH3hN2 or aH3aN2 (E and F). Virus in the cell culture supernatants at the indicated times post infection was titrated, and the titres were expressed as log10 (FFU/ml). Standard deviations are indicated. Significant differences were analysed using an unpaired two-tailed Student t test (*, P<0.05; **, P<0.01; ***, P<0.001; n.s., not significant).

To examine the contribution of the 2SBS to the HA-NA balance of virus particles we examined the replication phenotype of recombinant viruses containing either aN2 or the hN2 in the background of the 1968 pandemic virus A/Hong Kong/1/68 (H3N2) (referred to as hH3aN2 and hH3hN2) [28]. The hH3hN2 virus, lacking a functional 2SBS, produced large and clear plaques on Vero cells (Fig 3A and 3B, S6A and S6B Fig) as compared to the smaller, fuzzy plaques of the hH3aN2 virus with a functional 2SBS. Staining of plaques at 48 h post infection indicated that all cells within the plaques of hH3hN2 virus were infected, whereas many non-infected cells could be observed in the hH3aN2 plaques. This could be due to the more active aN2, which may destroy receptors on cells before the virus can enter into the cells. hH3aN2 reached lower titres than the hH3hN2 virus at 24 and 48 h post infection when Vero cells were used (Fig 3C), while no significant differences were observed for replication in MDCK cells (Fig 3D). Differences in cell surface sialosides and their distribution may explain differences between replication in Vero and MDCK cells. Although the sialylation patterns of MDCK and Vero cells are poorly characterized, both cell lines can be infected with human and avian IAVs and express α2,3- and α2,6-linked SIAs [49–51]. From these results we conclude that the absence or presence of a functional 2SBS in N2 may affect virus replication kinetics in a cell type-dependent manner.

### A functional 2SBS contributes to receptor binding of virus particles

**Fig 4:**
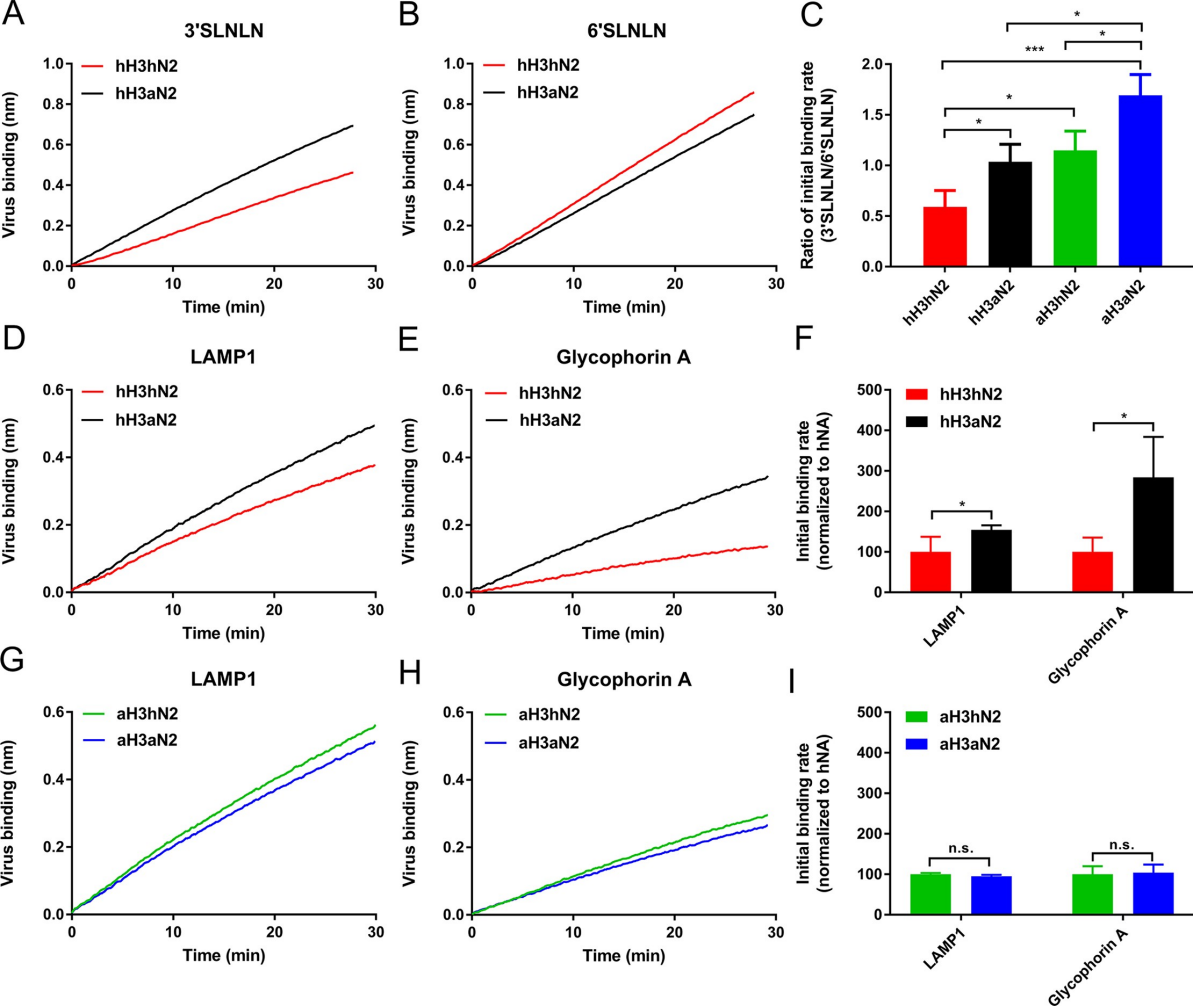
Binding of H3N2 viruses to LAMP1 and glycophorin. **A**. Identical number of hH3hN2 and hH3aN2 virus particles (determined using Nanoparticle Tracking Analysis; Nanosight NS300; S10 Fig) were analysed for their ability to bind 3’SLNLN (A), 6’SLNLN (B), LAMP1 (D) and glycophorin A (E) in the presence of OC using BLI. (C) Initial binding rates (v_obs_ = dB/dT) were determined as described previously [37] and ratios (3’SLNLN/6’SLNLN) were determined and graphed normalized to hH3aN2. Identical number of aH3hN2 and aH3aN2 virus particles were analysed for their ability to bind LAMP1 (G) and glycophorin A (H) in the presence of OC using BLI. Initial binding rates of the different virus-receptor combinations were determined and normalized to either hH3hN2 or aH3hN2 (F and I). Experiments were performed three times and representative experiments are shown (A, B, D, E, G, H). Mean values of these three independent experiments are shown (C, F, I). Standard deviations are indicated. Stars depict P values calculated using one-way ANOVA (C) or an unpaired two-tailed Student t test (F, I) (*, P<0.05; ***, P<0.001).

Using a recently established BLI-based kinetic binding assay [37] an enhanced initial binding rate to 3’SLNLN but not 6’SLNLN (Fig 4A and 4B), was observed for hH3aN2 virus containing a functional 2SBS in comparison to hH3hN2. As a result the hH3aN2 virus displayed a higher initial binding-rate ratio 3’SLNLN/6’SLNLN than hH3hN2 (Fig 4C, red and black bars). Next, two recombinant soluble glycoproteins containing mainly N-linked glycans (lysosomal-associated membrane glycoprotein 1 [LAMP1], ca. 18 N- and 6 O-linked glycans [52, 53]), or O-linked glycans (glycophorin A, ca. 16 O- and a single N-linked glycan [54, 55]) were used in BLI as recently described for recombinant fetuin [37]. LAMP1 and glycophorin A mimic the presumed functional and decoy receptors found on cells (LAMP1) and on mucins (glycophorin A) that are rich in N- or O-glycans, respectively. Analysis of the glycans on these glycoproteins by lectin binding using BLI confirmed the presence of sialylated N-linked glycans (both α2,3- and α2,6-linked) on both proteins, while only glycophorin A was shown to contain sialylated O-glycans (S7 Fig). Again, a functional 2SBS (present in aN2) contributed to virus binding (Fig 4D and 4E). This contribution was larger for binding to glycophorin A than to LAMP1 as judged from the initial binding rates (Fig 4F).

The HA of the 1968 pandemic H3N2 virus (referred to as hH3) prefers binding to terminal α2,6-linked SIAs [56, 57]. The results above implicate that, besides adaptations in HA, also adaptations in the 2SBS may contribute to a specificity-switch when an avian IAV adapts to humans. We therefore studied the effect of the 2SBS in NA when combined with an avian-type HA preferring binding to α2,3-linked SIAs. We generated the corresponding recombinant A/Hong Kong/1/68 (H3N2) viruses containing 7 amino acid substitutions in the HA (see S8A Fig). These substitutions reverted the HA back to the avian consensus sequence (referred to as avian-like aH3), including the crucial substitutions Q226L and G228S, which enable HA preferential binding to avian-type receptors [56, 57]. The resulting viruses are referred to as aH3hN2 and aH3aN2, depending on the absence and presence of the functional 2SBS, respectively. We confirmed the receptor-binding specificities of soluble hH3 and aH3 proteins by solid phase fetuin- and transferrin-binding assays and BLI (S8B and S8C Fig). As expected, aH3 displayed higher binding levels to fetuin, containing α2,3- and α2,6-linked SIAs, than hH3, while hH3 bound better than aH3 to transferrin, which only contains α2,6-linked sialoglycans. BLI analysis using H3-containing vesicles obtained from cells expressing full-length versions of hH3 or aH3 confirmed the different receptor-binding properties of these H3 proteins to 3’SLNLN and 6’SLNLN (S8D and S8E Fig). In contrast to viruses containing hH3, the presence of a functional 2SBS in aN2 enhanced replication of viruses with aH3 both on Vero (Fig 3E) and MDCK (Fig 3F) cells. Differences in virus replication were smaller for MDCK than for Vero cells. We next analysed receptor-binding properties of aH3hN2 and aH3aN2 viruses using BLI. As observed before for the hH3-containing viruses (Fig 4C; red and black bars), a functional 2SBS enhanced binding to 3’SLNLN but not 6’SLNLN when N2 was combined with aH3 (Fig 4C). However, viruses containing aH3 displayed similar binding kinetics in the presence of OC regardless of the presence of a functional 2SBS for both LAMP1 and glycophorin A (Fig 4G, 4H and 4I). From these results we conclude that a functional 2SBS site in NA contributes to virion-receptor binding in a HA- and receptor-dependent manner.

### Substrate binding by NA and HA both affect enzymatic cleavage by NA in virus particles

**Fig 5:**
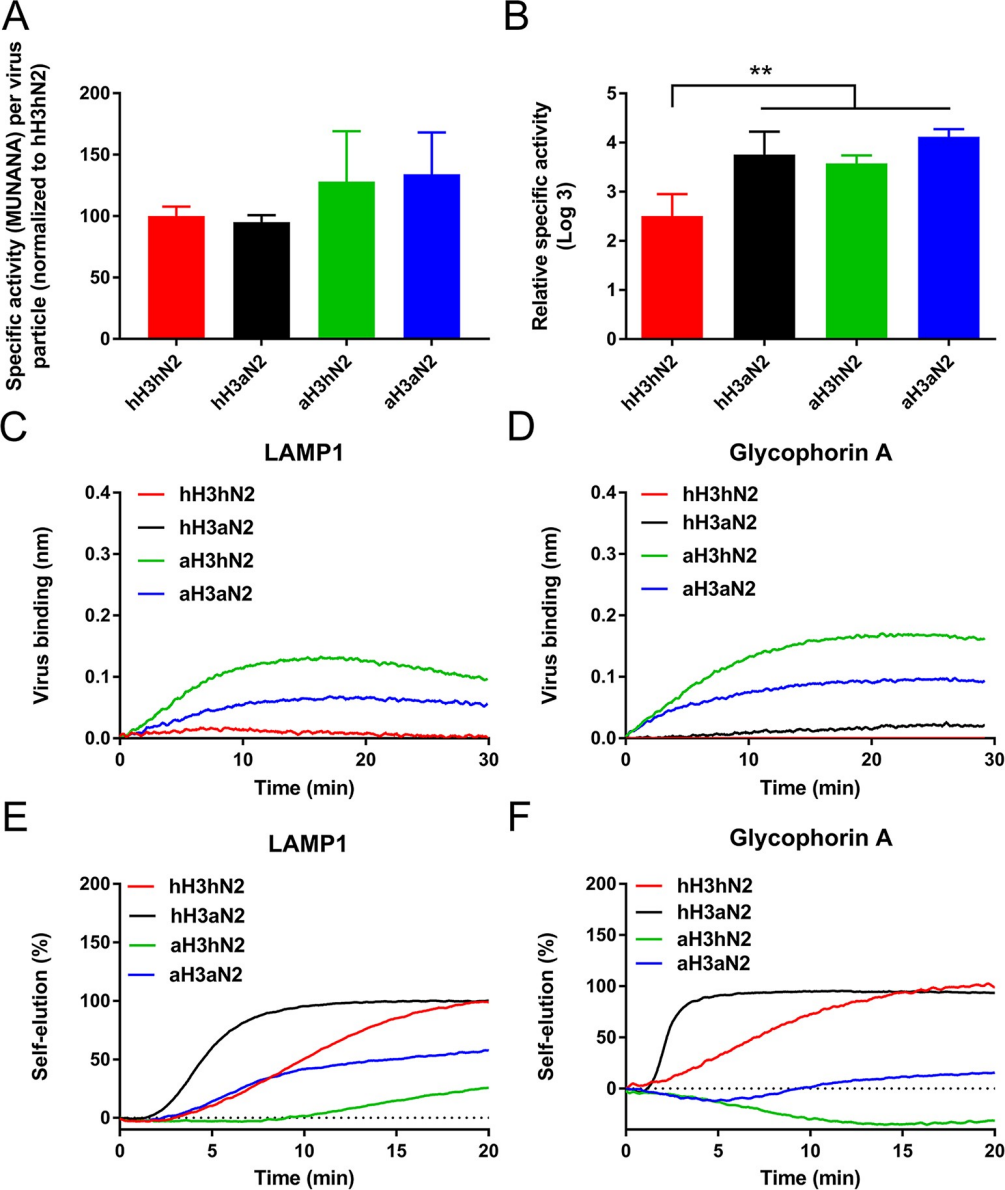
NA enzymatic activity in virus particles. (A) Numbers of particles in virus preparations were determined using Nanoparticle Tracking Analysis (Nanosight NS300); the NA activity in these preparations was analysed by MUNANA assay. Relative NA activity per virion is graphed (N = 3). (B) Virus preparations were normalized based on their MUNANA activity. Fetuin-coated plates were incubated with serial dilutions of viruses in the absence of OC. Cleavage of SIAs from glycoproteins was monitored using ECA, which binds desialylated glycans. Dilutions corresponding to half-maximum lectin binding were determined by non-linear regression analysis and used to calculate the specific activity of the different viruses. See S9 Fig for curves. Mean values of two independent experiments performed in duplicate are shown. Standard deviations are indicated. Stars depict P values calculated using one-way ANOVA (**, P<0.01). (C and D) Identical number of hH3hN2 and hH3aN2, and aH3hN2 and aH3aN2 virus particles were analysed for their ability to bind LAMP1 (C) or glycophorin A (D) in the absence of OC using BLI. (E and F) After binding of virus preparations with identical particle numbers to LAMP1 (E) or glycophorin A (F) in the presence of OC (similarly as shown in Fig 4), OC was removed by three repeated washes and virion self-elution in the absence of OC was monitored. Dissociation of virus particles was normalized to the virus association levels in the presence of OC. Experiments were performed three times. Representative experiments are shown.

The NA enzymatic activity of the different recombinant viruses with and without a functional 2SBS was analysed using the monovalent soluble substrate MUNANA, by ELLA and by BLI. The different viruses displayed a similar NA activity per particle using the monovalent soluble substrate MUNANA (Fig 5A). As also the NA proteins do not differ in their MUNANA activity regardless of the presence or absence of a functional 2SBS (Fig 1A), we conclude that similar amounts of NA are incorporated into virions of the four viruses. The viruses differed, however, in their specific activities when the multivalent glycoprotein fetuin was used as substrate in an ELLA (Fig 5B). hH3hN2 virus was less active compared to viruses containing aN2 and/or aH3, indicating a contribution of receptor binding via HA and the 2SBS to NA enzymatic activity in the context of virus particles. In agreement with the results obtained with the recombinant proteins (Fig 1B), cleavage of α2,6-linked SIA found on transferrin was less efficient and did not appear to differ significantly between the different viruses (S9 Fig).

The ELLAs (Fig 5B and S9 Fig) indicate that both receptor binding via HA and the 2SBS of NA contribute to the sialidase specific activity of virus particles. These endpoint assays do not, however, elucidate the HA-NA balance of these viruses, for which kinetic BLI assays are required [37]. Preliminary experiments showed inefficient cleavage of the synthetic glycans by the recombinant viruses. Kinetic assays to determine the HA-NA balance of these viruses were therefore performed with the glycoprotein receptors (LAMP1 or glycophorin A). In the absence of OC, that is, with active NA proteins, no appreciable binding of hH3-containing viruses could be detected indicating efficient receptor cleavage by NA (Fig 5C and 5D). Limited binding could be detected, however, for the aH3-containing viruses in the absence of OC. The binding curve of the virus with a functional 2SBS (aH3aN2) bended earlier and had a smaller area under the curve than that of the virus without a functional 2SBS (aH3hN2) for both LAMP1 and glycophorin A. This bending of the curves is explained by ongoing cleavage of SIAs by viruses attached to the sensor-attached glycoproteins, resulting in release of bound virus particles [37]. The earlier bending and smaller area under the curve observed for the aH3aN2 virus is indicative of more efficient cleavage of the sensor-attached receptors by this virus than by aH3hN2, lacking a functional 2SBS.

The effect of receptor binding via NA and HA on NA activity of virions was analysed further by NA-dependent virion self-elution from a receptor-coated BLI sensor after prior binding of the virions in the presence of OC. Self-elution of IAV particles requires NA activity and self-elution is not observed when NA activity is blocked by OC [37]. After binding of the four recombinant viruses to LAMP1 and glycophorin A in the presence of OC, OC was removed by repeated short washes in Dulbecco’s phosphate buffered saline (PBS) with Calcium and Magnesium and virus self-elution was monitored. Clearly, viruses with aN2 proteins eluted faster from the sensors than the viruses with hN2 (compare hN3aN2 with hN3hN2 and aH3aN2 with aH3hN2; Fig 5E and 5F), for both glycoprotein receptors. Of note, NA-depended self-elution of virus particles is often preceded by an apparent increase in virus binding [37] represented here as negative self-elution, particularly in the case of aH3aN2 and aH3hN2 (Fig 5F). The larger negative area of self-elution for aH3hN2 reflects the reduced NA activity of this virus compared to aH3aN2. Also the identity of HA affected the virus self-elution rate. Viruses with hH3 eluted faster than corresponding viruses with aH3 (e.g. compare hH3aN2 with aH3aN2). For hH3aN2, self-elution was faster from glycophorin A than from LAMP1. For aH3-containing viruses, the opposite was observed. Differences in virion self-elution observed for different HA-receptor combinations could be due the different receptor repertoires present on the two proteins (S7 Fig). The results indicate that receptor binding via the 2SBS of NA contributes to enzymatic cleavage by NA in virions and to virion self-elution from a receptor-coated surface. Virion self-elution was also shown to depend on the identity of the HA and the glycoprotein receptor used.

### The 2SBS in 1968 pandemic H3N2

**Fig 6:**
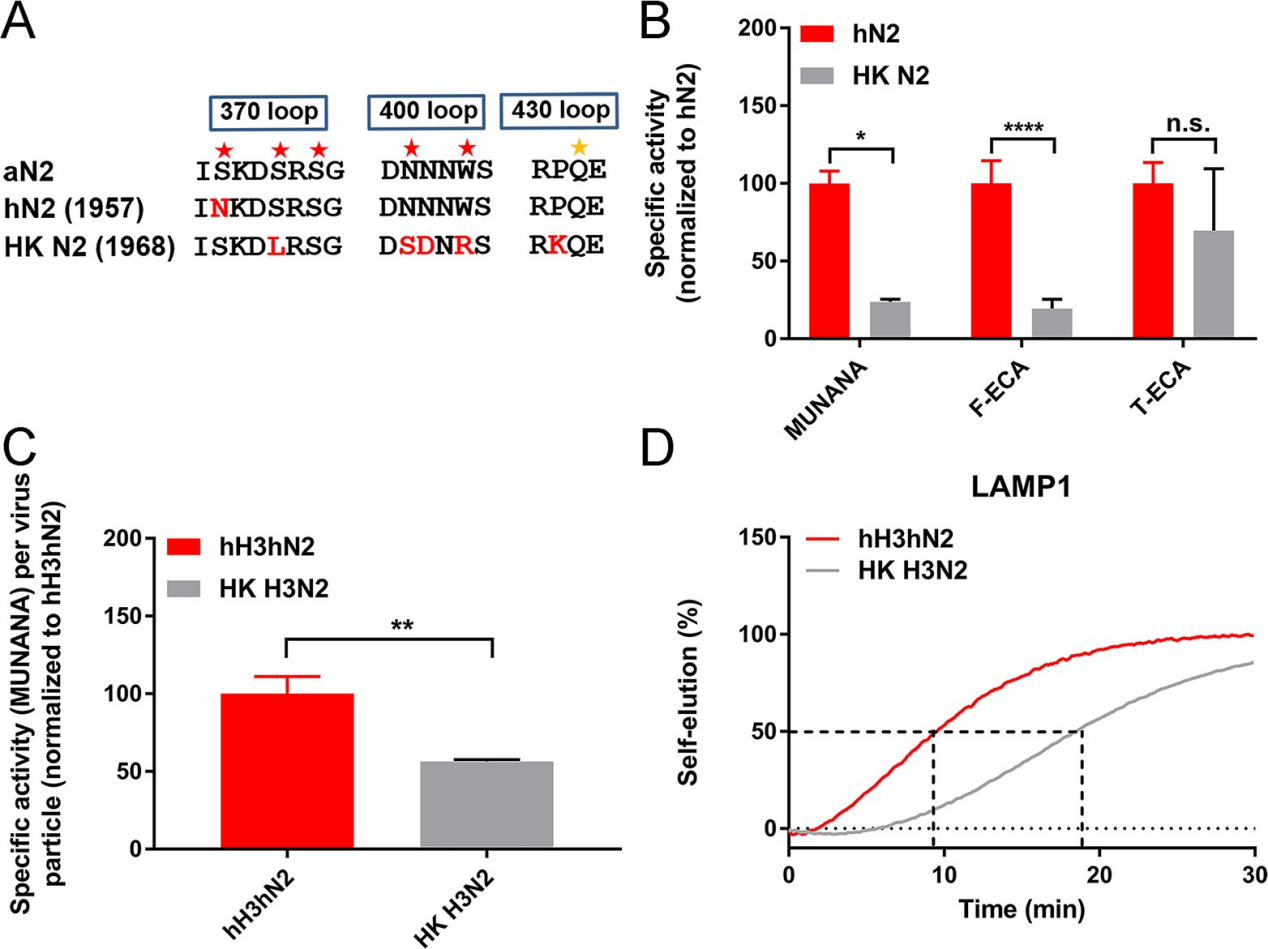
Enzymatic activity of HK N2 protein. (A) Sequences of the three loops that make up the 2SBS of aN2, hN2 and HK N2 are shown. Red asterisks indicate SIA-contact residues in N2. The orange asterisk indicates an additional SIA-contact residue in the 430 loop of N9. Residues that differ from the aN2 sequence, which corresponds to the avian N2 consensus sequence, are shown in red. (B) Specific activity of hN2 and HK N2 recombinant soluble proteins as determined using the MUNANA assay or ELLA using the Fetuin-ECA (F-ECA) combination are graphed relative to hN2. (C) Relative NA activity (determined by MUNANA assay) per virus particle is graphed for hH3hN2 and HK H3N2 viruses. (B and C) Mean values of three independent experiments are shown. Standard deviations are indicated. Stars depict P values calculated using an unpaired two-tailed Student t test (*, P<0.05; **, P<0.01; ****, P<0.0001). (D) BLI analysis of virion self-elution from LAMPI-coated sensors was analysed for hH3hN2 and HK H3N2 virus particles. Prior to self-elution in the absence of OC, viruses were bound to similar levels to LAMP1 in the presence of OC. The experiment was performed three times, a representative experiment is shown.

The S367N mutation in the 2SBS of N2 was rapidly obtained after emergence of the H2N2 pandemic virus in 1957 and was observed in human H2N2 viruses until 1958. Most viruses isolated thereafter did not contain the S367N mutations but rather contained the S370L mutation, which also results in loss of a SIA-contact residue in the 370 loop (S3 Fig) and hemadsorption activity [28]. Both single mutations had a similar negative effect on catalytic activity of the 1957 NA [28]. These results indicate that there was not a selection against S367 per se, but rather against a functional 2SBS, which is achieved by either mutation. However, several additional mutations accumulated in time in the three loops of the 2SBS. N2 from the A/Hong Kong/68 (H3N2) (referred to as HK N2) contains five mutations (S370L, N400S, N401D, W403R, P432K) in the 2SBS compared to the avian consensus sequence (Fig 6A). To analyse the contribution of the 2SBS to the enzymatic activity of these different N2 proteins, a comparative analysis of recombinant proteins and viruses using monovalent and multivalent substrates was performed. The HK N2 protein was 4–5 fold less active than hN2 both on the monovalent substrate MUNANA and the multivalent substrate fetuin. Cleavage of sialoglycans attached to transferrin was not significantly affected (Fig 6B). We also compared the NA activity of recombinant H3N2 viruses only differing in their NA segment. HK H3N2 virus, containing the 1968 HK N2 protein, displayed 2-fold lower NA activity per virus particle than the hH3hN2 virus, containing the 1957 N2 protein, as determined by MUNANA cleavage (Fig 6C). Similarly, the time required for 50% self-elution of virions from the multivalent receptor LAMP1, was 2-fold longer for HK H3N2 than for hH3hN2 (Fig 6D). Thus, hN2 from 1957 and 1968 HK N2 differ to a similar extent in their catalytic activity both when monovalent or multivalent substrates are used. As receptor-binding via the 2SBS only increases NA activity for multivalent, but not monovalent substrates, we conclude that these differences do not result from differences in receptor-binding by their (non-functional) 2SBS. Moreover, the difference observed when comparing the two recombinant proteins is similar to the difference in activity of the two NAs in the virus context.

## Discussion

Since the discovery of hemadsorption activity in NA 1984 [58] and the structural evidence of the 2SBS in N9 1997 [29], only few studies have addressed 2SBS-mediated receptor binding and the functional consequences thereof for NA activity [26, 28, 33, 34, 59]. We now show that the 2SBS is an important factor in the complex interplay between HA, NA and receptors, referred to as the HA-NA-receptor balance. A functional 2SBS in N2 was shown to prefer binding to α2,3-linked sialosides similarly to N1 [26] and N9 [33]. In agreement herewith, it enhances catalytic activity against substrates carrying α2,3-linked SIAs. The contribution of the 2SBS to the HA-NA-receptor balance of virus particles was shown to be receptor- and HA protein-dependent as demonstrated by kinetic analysis of receptor-binding and -cleavage of virions using BLI. The 2SBS was shown to contribute to receptor binding also when NA was combined with a receptor-binding HA in IAV virions, as well as to cleavage of receptors by virions and to virion self-elution from a receptor-coated surface. The absence or presence of a functional 2SBS also affected virus replication in a cell type- and HA-dependent manner. Our results indicate that mutation of the 2SBS as observed in early human pandemic viruses negatively affects the catalytic activity of NA and may serve to restore the HA-NA-receptor balance of viruses carrying HA proteins with altered receptor-binding properties in relation to a novel host sialome. Conservation of the 2SBS in most avian strains, with the notable exception of H9N2 viruses, is lost in human [26, 29, 30, 34], swine and canine variants (S2 Fig). Strong conservation usually reflects a critical function. It would be very interesting to investigate in depth whether a critical function for the 2SBS in avian strains, for instance related to the HA-NA-receptor balance, is not required for efficient replication and transmission of human, canine and swine strains.

N2 prefers binding of α2,3- over α2,6-linked SIAs via its 2SBS. The specificity of the N2 2SBS correlates with the enhanced cleavage of substrates carrying α2,3-linked SIAs compared to substrates carrying only α2,6-linked sialosides. Of note, enhanced activity was also observed for α2,6-linked SIAs at least when these sialosides were linked to substrates additionally carrying α2,3-linked SIAs (Fig 1A; fetuin-SNA combination). These results indicate that the 2SBS enhances catalytic activity by bringing sialosides on multivalent substrates close to the catalytic site and that, depending on the substrate used, the enhanced cleavage of SIAs not necessarily matches the specificity of the 2SBS. Preferred binding of avian-type receptors via its 2SBS was previously also observed for N9 [33] and N1 [26], suggesting that this is a conserved feature for NAs of different subtypes. We cannot exclude, however, that the 2SBS of different NA subtypes may differ in their receptor-binding fine specificity, as structural differences were observed in the interactions between ligands and the 2SBS for different NA subtypes [30]. In N9, the conserved K432 residue in the 2SBS forms a hydrogen bond with SIA [29] and mutation K432E in N1 has a large negative effect on the cleavage of multivalent substrates [26]. In contrast, several other avian NA subtypes, including N2, contain a Q or E residue at this position, which does not form a hydrogen bond with SIA in the few available crystal structures [30]. Previously, it was shown that N1 and N2 NAs bound with similar efficiency to both avian and human type receptors SIAs [28, 35]. This discrepancy is probably explained by the different methods used to analyse the receptor specificity of the 2SBS. In the previous reports, a red blood cell binding assay was employed, in which desialylation of erythrocytes was followed by resialylation using α2,3- or α2,6-sialyltransferases. Binding to resialylated erythrocytes might be affected by prior incomplete desialylation. Alternatively, a higher receptor density on erythrocytes compared to the BLI sensor surface might allow for binding of α2,6-linked SIAs. The ability of the 2SBS to bind human-type receptors to some extent is also suggested by the modestly increased or decreased cleavage of SIAs from substrates only containing α2,6-linked SIAs upon the introduction of mutations in the 2SBS (this study and [26, 28]).

The 2SBS contributed to receptor-binding also when NA was combined with a receptor-binding HA in IAV virions. In combination with HA preferring binding to α2,6-linked SIAs (hH3), the 2SBS enhanced binding for all receptors analysed, except 6’SLNLN, to which the recombinant aN2 protein did not bind. Binding to glycophorin A, carrying many O-linked sugars also found on mucins, was more enhanced by the 2SBS than binding to LAMP1, which carries mostly sialylated N-glycans. The functional significance of this difference remains to be determined. When combined with HA that prefers binding to α2,3-sialosides (aH3), the enhancing effect of the 2SBS was not observed for the glycoprotein receptors analysed. Thus, the contribution of NA to virion-receptor binding depends on the specificity/affinity of the corresponding HA and the receptors present. Previously it was shown that the active site of NA contributes to virion-receptor binding in case of a low-activity catalytic site [37], a characteristic which is also appears to be displayed by recent H3N2 viruses [60, 61]. As we now show that a functional 2SBS in NA can also contribute to virion-receptor binding, two mechanisms exist by which NA can assist in binding of virions to host cells.

A complex interplay between HA, NA and receptor determines the attachment of virus particles to and release from a receptor-containing surface. This HA-NA-receptor balance can be experimentally determined using kinetic BLI assays by analysis of virus binding in the absence or presence of NA inhibitors and self-elution from different receptors (this paper and [37]). The HA-NA-receptor balance determines the residence time of a virus on a sialylated surface and the speed by which it moves over this surface. We assume that an optimal balance is important for virions to efficiently pass the heavily sialylated mucus layer, while still allowing virion attachment to host cells resulting in endocytic uptake. The complexity of the HA-NA-receptor balance is exemplified by the contribution of NA to receptor binding [37] and of HA to the apparent catalytic activity of NA (this paper)[37, 62]. We now show that the HA-NA-receptor balance as reflected for example in virion self-elution (Fig 5) is affected by a functional 2SBS, depending on the particular HA with which NA is combined and the receptors used. Changes in the 2SBS of NA should thus be considered in the context of mutations affecting the receptor-binding site of HA and the catalytic site of NA.

The 2SBS of N2 appears to accumulate more mutations than other surface exposed parts of the NA protein (S3 Fig). While the 1957 N2 protein has a single substitution in the 2SBS, the 1968 N2 protein contains five mutations in this site. The accumulation of several mutations in the 2SBS was found to have no further negative effects on the enzyme-enhancing function of 2SBS as compared to a single mutation of a SIA contact residues in the 2SBS of an early pandemic virus from 1957. Although we cannot exclude that the accumulation of mutations in the 2SBS of N2 indicates ongoing adaptation of NA to the human host or serves to restore subtle deviations in the HA-NA-receptor balance resulting from other mutations in HA and/or NA, it seems more likely that it rather results from continuous immune pressure on this site [22, 63] in combination with loss of functional importance of the 2SBS in human viruses.

An important role for the NA 2SBS in IAV replication *in vivo* is suggested by the conservation of this site among NA subtypes of most avian viruses, the rapid loss of this site in human pandemic viruses ([1, 26, 28, 30, 36] and S2 Fig), the important role of this site in HA-NA-receptor balance (this study) and observations that this site affects virus replication *in vitro* ([26, 34, 59] and this study). Of note, we now show that the presence or absence of a functional 2SBS affected virus replication depending on the receptor-binding properties of HA, with which NA was combined. Replication of viruses with a human or avian-like HA is enhanced by the absence or presence of a functional 2SBS, respectively, although some cell-dependent differences were observed. The absence or presence of a functional 2SBS was reported not to affect influenza viral replication in ducks [34]. However, in this latter study recombinant viruses were used containing HA from a H2N9 and NA from a H3N2 virus. This may have resulted in a mismatched HA-NA combination in which the presence of the 2SBS might be of minor influence on replication. Alternatively, the 2SBS may be important for virus transmission rather than for replication in ducks *per se*. Clearly, additional experiments are needed to demonstrate the importance of the 2SBS for IAV replication and transmission *in vivo*. Interestingly, both for H9N2 and H7N9 viruses, the well-known Q226L mutation in the receptor-binding site of HA, resulting in a shift from avian to human receptor specificity, is associated with mutations in the 2SBS that negatively affect receptor binding [33, 36]. These avian viruses thus display a striking parallel with the changes observed in the receptor-binding sites of HA and NA of avian-origin pandemic viruses. We propose that mutations in the 2SBS of avian viruses may be indicative of an as of yet underappreciated, increased potential of avian viruses to cross the host species barrier. Of note, also upon introduction of coronavirus OC43 into humans, the lectin function of the receptor-destroying hemagglutin-esterase protein was lost through progressive accumulation of mutations resulting in reduced cleavage of multivalent substrates [64]. Thus, both coronaviruses and IAVs appear to adapt to the sialoglycome of the human respiratory tract by tuning the virion receptor-binding and cleavage functions, the latter among others by mutation of the lectin domain of the receptor-destroying NA.

## Materials and methods

### Expression of recombinant proteins

Human-codon optimized cDNAs (Genescript) encoding the N2 ectodomain of A/Singapore/1/57(H2N2) (GenBank accession no. AY209895.1; referred to as human N2 [hN2]) and a variant thereof containing the N367S mutation (referred to as avian-like N2 [aN2]) were cloned into a pFRT expression plasmid (Thermo Fisher Scientific) in frame with sequences encoding a signal sequence derived from Gaussia luciferase, a Strep tag and a Tetrabrachion tetramerization domain, similarly as described previously [20]. The corresponding full length (FL) NA-coding plasmids were generated by replacement of the non-NA coding sequences by sequences encoding the NA transmembrane domain and cytoplasmic tail of N2 of A/Singapore/1/57(H2N2). Human-codon optimized cDNAs encoding FL H3 or the H3 ectodomain of A/Hong Kong/1/68 (H3N2) (GenBank accession no. CY033001; referred to as human H3 [hH3]) or of an variant thereof containing 7 amino acid substitutions, which revert the HA back to the avian consensus sequence [56] (referred to as avian-like H3 [aH3]) were cloned in pCD5 expression vectors similarly as described previously [65]. Codon optimized glycoproteins LAMP1 and glycophorin A ectodomain-encoding cDNAs (Genescript) were genetically fused to Fc-tag, for Protein-A based purification, and a Bap tag [66], for binding to octet sensors, and cloned in a pCAGGs vector, similarly as described previously for fetuin [37]. NA and glycoprotein expression plasmids were transfected into HEK293T (ATCC) cells using polyethylenimine (PolyScience) [20]. An expression vector encoding BirA ligase was cotransfected with the LAMP1- and glycophorin A-coding vectors [37]. Five days post transfection, cell culture media containing soluble NA proteins and glycoproteins were harvested and purified using Strep tactin or protein A containing beads [20, 37]. Purified NA proteins were quantified by quantitative densitometry of GelCode Blue (Thermo Fisher Scientific)-stained protein gels additionally containing bovine serum albumin (BSA) standards. The signals were imaged and analysed with an Odyssey imaging system (LI-COR). HEK293T cells were transfected with full-length NA constructs to obtain membrane vesicles. To this end, cells were vesiculated as described previously [26, 48]. VLPS and membrane vesicle preparations were purified using Capto Core 700 beads (GE Healthcare Life Sciences) according to the manufacturer’s instructions and as detailed previously [67] to remove proteins smaller than 700 kDa. The amount of NA protein in the VLPs and vesicle preparations was determined using the MUNANA assay described below.

### Viruses

Generation of recombinant virus HK H3N2, which harbours all genes from the pandemic virus A/Hong Kong/1/68 (H3N2) has been described before [57]. Also the generation of hH3hN2 and hH3aN2 viruses, which carry the N2 gene of the pandemic A/Singapore/1/1957 (H2N2) in the background of A/Hong Kong/1/68 (H3N2) has been described before [28]. The hH3aN2 virus contains substitution N367S in the N2 protein. aH3hN2 and aH3aN2 viruses were generated as described previously [56] in the background of A/Hong Kong/1/68 (H3N2). These latter viruses carry the H3 protein of A/Hong Kong/1/68 (H3N2) containing 7 amino acid substitutions in HA which revert the HA back to the avian consensus sequence [56] combined with the N2 protein of A/Singapore/1/1957 (H2N2) with (aH3aN2) or without (aH3hN2) the N367S substitution. Virus stocks were grown in MDCK-II cells (ECACC). Viruses were inactivated by UV radiation using UV Stratalinker 1800 (Stratagene) on 50,000 μJoules prior to their use in the binding and cleavage assays. UV inactivation did not affect the enzymatic activity of NA as determined with the MUNANA assay.

### NA cleavage assays

The NA enzymatic activity was determined by using a fluorometric assay [68] in combination with 2’-(4-Methylumbelliferyl)-α-D-N-acetylneuraminic acid (MUNANA; Sigma-Aldrich) as described previously [20]. Enzymatic activity of the NA proteins towards multivalent glycoprotein substrates was analysed using a previously described enzyme-linked lectin assay (ELLA) [33]. In brief, fetuin- or transferrin-coated plates were incubated with serial dilutions of recombinant soluble NA proteins. After overnight incubation at 37°C, plates were washed and incubated with either biotinylated *Erythrina Cristagalli* Lectin (ECA, 1.25 μg/ml; Vector Laboratories), biotinylated peanut agglutinin (PNA, 2.5 μg/ml; Galab Technologied), biolinylated *Sambucus Nigra* Lectin (SNA, 1.25 μg/ml; Vector Laboratories) or biotinylated *Maackia Amurensis* Lectin I (MAL I, 2.5 μg/ml; Vector Laboratories). Cleavage of SIAs from fetuin and transferrin was quantified by analysing the increase (PNA and ECA) or decrease (MAL I and SNA) in binding of different lectins depending on their binding specificities (S4 Fig). The binding of ECA, PNA, SNA and MAL I was detected using horseradish peroxidase (HRP)-conjugated streptavidin (Thermo Fisher Scientific) and tetramethylbenzidine substrate (TMB, bioFX) in an ELISA reader EL-808 (BioTEK) by measuring the optical density (OD) at 450 nm. The data were fitted by non-linear regression using the Prism 6.05 software (GraphPad). The resulting curves were used to determine the amount of NA protein corresponding to half maximum MUNANA cleavage or lectin binding. The inverse of this amount is a measure of specific activity (activity per amount of protein) and was graphed relative to other NA proteins or substrate-lectin combinations.

### Plaque assay

Plaque assays were performed in Vero cells (ATCC) as described previously [69]. One hour after infecting the cell monolayers with 30–50 plaque forming units of the virus in 1 ml of maintenance medium, the virus inoculum was removed and cells were covered the Avicel RC-581 overlay medium and cultures were incubated at 37°C in 5% CO2 atmosphere. After three days of incubation, the overlay was removed by suction and the cells were fixed with 10% formalin and stained with 1% crystal violet solution in 20% methanol in water. For immunostaining, cells were fixed with 4% paraformaldehyde solution for 30 min at 4°C, washed with PBS and permeabilized by incubation for 10–20 min with buffer containing 0.5% Triton-X-100 and 20 mM glycine in PBS. Cell layers were incubated with monoclonal antibodies specific for the influenza A virus nucleoprotein (kindly provided by Dr. Alexander Klimov at Centers for Disease Control, USA) for 1 hour followed by another 1 hour incubation with peroxidase-labeled anti-mouse antibodies (DAKO, Denmark) and 30 min incubation with precipitate-forming peroxidase substrates True Blue. Stained plates were washed with water to stop the reaction, scanned on a flatbed scanner and the data were acquired by Adobe Photoshop 7.0 software.

### Virus replication in Vero or MDCK cells

To characterize replication kinetics of different recombinant viruses, two replicate cultures of Vero or MDCK cells in 12-well plates were infected with each virus at MOI 0.001 (Vero cells) or 0.0001 (MDCK cells). Inocula were removed 1 hpi, fresh medium was added, and cultures were incubated at 37°C. Samples of culture supernatant were taken 24, 48 and 72 hpi and stored frozen. They were titrated together using focus formation assay in MDCK cells as described previously [57]. Numbers of infected cells per well were counted for the virus dilution that produced from 30 to 300 infected cells per well and recalculated into numbers of focus forming units (FFU) per ml of the original undiluted virus suspensions.

### Biolayer interferometry (BLI) binding and cleavage assays

For the full length protein-containing vesicles and VLPs, similar amounts of NA activity, and thus NA protein, were applied in the BLI assays using the Octet RED348 (Fortebio). Inactivated virus preparations were analysed using Nanoparticle Tracking Analysis (Nanosight NS300, Malvern) as detailed below in order to use similar number of virus particles in the BLI assays. BLI assays were performed as described previously [37]. All experiments were carried out in Dulbecco's PBS with Calcium and Magnesium (Lonza) at 30°C and with sensors shaking at 1000 rpm. Streptavidin biosensors were loaded to saturation with biotinylated synthetic glycans 2,3-sialyl-N-acetyllactosamine-N-acetyllactosamine (3’SLNLN), 2,6-sialyl-N-acetyllactosamine-N-acetyllactosamine (6’SLNLN), N-acetyllactosamine-N-acetyllactosamine (LNLN), LAMP1 or glycophorin A glycoproteins. Synthetic glycans were synthesized at the Department of Chemical Biology and Drug Discovery, Utrecht University, Utrecht, the Netherlands. For the NA kinetic cleavage assay, the sensors loaded with synthetic glycans were incubated in 100 μl buffer containing 4 μg recombinant soluble aN2 or hN2 in the absence or presence of 8 μg ECA. As controls, sensors were also incubated with ECA in the absence of N2. Association of the N2 VLPs, vesicles and virus particles was analysed for 30 minutes in the absence or presence of 10 μM OC (Roche). For viruses, the virus association phase in the presence of OC was followed by three 5 s washes and a dissociation phase in the absence of OC. Initial binding rates were determined similarly as previously described [37]. For lectin binding, the sensors loaded with recombinant glycoproteins were incubated with the different lectins (8 μg/100 μl) for 15 minutes.

### Nanoparticle tracking analysis (NTA)

NTA measurements were performed using a NanoSight NS300 instrument (Malvern) following the manufacturer’s instructions. The UV-inactivated virus preparations were diluted with PBS to reach a particle concentration suitable for analysis with NTA. All measurements were performed at 19°C. Per analysis, the NanoSight NS300 recorded five 60 second sample videos, which were then analysed with the Nanoparticle Tracking analysis 3.0 software, resulting in quantitative information on particle number and particles sizes (S10 Fig). Each virus preparation was analysed twice and mean values were used. NTA measurements were validated by analysis of virus stocks quantified earlier by silver staining of viral proteins after electrophoresis on polyacrylamide gels [37]. Results obtained via both methods correlated well (less than 25% deviation).

## Supporting information

S1 Fig N2 Crystal structure. (A) Surface representation of the crystal structure of the N2 from pandemic A/RI/5 +/1957 (H2N2) in complex with Neu5Ac (PDB ID:4H53; [70]) was depicted using Pymol software. Top view is shown. The SIA-contact residues in the NA active site and the 2SBS are coloured green and red, respectively. The Neu5Ac moieties in the 2SBS sites are shown in a stick representation.
(TIF)

S2 Fig Sequence logos of the 2SBS of N2 proteins of viruses infecting different species. Sequence logos were generated for the three loops (370, 400 and 430 loop) that constitute the 2SBS using DNASTAR Lasergene 14 software (MegAlign Pro 14). The overall height of the stack indicates the sequence conservation at that position, while the height of symbols within the stack indicates the relative frequency of each amino acid at that position. All sequences available for avian viruses containing N2 excluding H9N2 (indicated by Avian HxN2), avian H9N2, dog H3N2, human H2N2, human H3N2 until 2000, swine H3N2 and swine H1N2 from the Influenza Research Database (https://www.fludb.org/) were used. SIA-contact residues were highly conserved in Avian HxN2, but not in H9N2 viruses. Avian H9N2 viruses were mainly (>80%) found in Galliformes species (chicken, turkey and quail), while avian HxN2 viruses were isolated mainly from non-Galliformes species (>75%). Dog H3N2 viruses generally contain a S370L mutation in the 370 loop, which is known to affect functionality of the 2SBS [28], while in addition the identity of the 430 residue deviates from those found in avian viruses. Please note that the phylogenetic analysis shown in S3 Fig indicates that human H2N2 viruses either have a mutated SIA-contact residue at position 367 or at position 370, both of which are known to disrupt the 2SBS [28]. Swine viruses containing N2, which are generally derived from human viruses [71], also contain a mutated 2SBS. SIA-contacting residues were labelled with asterisks in the sequence logo of the avian HxN2 viruses. The grey asterisk indicates an additional SIA-contact residue in the 430 loop of N9. Numbering of the start and end residues of the three loops is indicated.
(TIF)

S3 Fig Phylogenetic analysis of N2 of human H2N2 and H3N2 viruses from 1957 until 1980. All full-length and unique N2 protein sequences of human H2N2 and H3N2 viruses between 1957–1980 were downloaded from the GenBank and GISAID databases. N2 protein trees were constructed by using the PHYLIP neighbor-joining algorithm with the mPAM distance matrix. This tree was used as a guide tree to select N2 sequences representing all main branches of the tree. The selected N2 proteins were used to construct a summary tree with topology similar to that of the guide tree. Mutations that became fixed along the trunk of the tree are indicated as well as 2SBS residues that differ between different branches. On the right site the residues of the 370, 400 and 430 loops that make up the 2SBS are shown. SIA-contact residues in the N2 protein are indicated by the red shading. Mutations in N2 relative to the avian consensus sequence are shown in red.
(TIF)

S4 Fig Enzymatic activity of N2 proteins using monovalent and multivalent substrates. (A) The enzymatic activity of hN2 and aN2 proteins for a monovalent substrate was determined using the MUNANA fluorometric assay. To this end, limiting dilutions of the different N2 proteins were subjected to the assay and the fluorescence generated upon cleavage of MUNANA was measured using a plate reader (in relative fluorescent units [RFU]). The data were fitted by non-linear regression using the Prism 6.05 software (GraphPad). The resulting curves were used to determine the amount of NA protein corresponding to half maximum MUNANA cleavage (indicated by the arrow). The inverse of this amount is a measure of specific activity (activity per amount of protein) and was graphed relative to hN2 in (B). ELLAs were used to determine the relative specific activities of the N2 proteins for multivalent substrates (C-F). The OD 450nm values correspond to lectin binding upon incubation of the glycoprotein with different dilutions of the NA preparations. In the examples shown, removal of SIAs from fetuin was probed using the lectins ECA (C) and MAL I (E). Increasing dilutions of the NA preparations resulted in reduced cleavage of SIAs as indicated by the reduced binding of ECA, which (just as PNA) binds to desialylated glycans. The opposite was observed for MAL I (and SNA) which binds to sialylated glycans. The data were fitted by non-linear regression using the Prism 6.05 software (GraphPad). The resulting curves were used to determine the dilution (or amount) of NA protein corresponding to half maximum lectin binding (indicated by the arrow). This value was used to determine the relative specific activity (activity per amount of protein) for a specific glycoprotein-lectin combination (D and F).
(TIF)

S5 Fig Hemagglutination assays. (A) Identical amounts of recombinant soluble hN2 and aN2 protein were pre-complexed with a strepMabClassic-HRP and rabbit-α-mouse-HRP prior to their incubation with erythrocytes. Serial twofold dilutions of the antibody-N2 complexes were incubated with equal volumes of 0.5% human erythrocytes at 4°C for 2 h in the presence of OC. Red dots at the bottom of the wells indicate absence of hemagglutination. (B-C) Hemagglutination using membrane vesicles (B) or VLPs (C) containing identical amounts of N2 protein. Membrane vesicles containing full length hN2 and aN2 were analysed for their ability to bind 3’SLNLN (D) or 6’SLNLN (E) in the absence or presence of OC using BLI similarly as described in the legend to Fig 2. Representative experiments (out of three performed) are shown.
(TIF)

S6 Fig Plaque morphology of recombinant viruses. Plaque assays were performed for hH3hN2 (A) and hH3aN2 (B) viruses using Vero cells followed by crystal violet dye staining.
(TIF)

S7 Fig Analysis of glycans attached to glycophorin A and LAMP1 using lectin binding. Glycophorin A and Lamp1 were analysed for their attached glycans by BLI analysis of lectin binding to sensors coated with these glycoproteins. As a negative control, empty sensors were used. (A) Binding of MAL I, which is specific for SIAα2,3Galα1,4GlcNAc oligosaccharide abundantly present on N-linked glycans. (B) Binding of MAL II, which is specific for SIAα2,3Galβ1,3GalNAc oligosaccharides abundantly present on O-linked glycans. (C) Binding of SNA, which is specific for SIAα2,6Galβ1,4GlcNAc oligosaccharide abundantly present on N-linked glycans. (D) Binding of ECA, which is specific for terminal Galβ1,4GlcNAc glycans corresponding to non-sialylated N-linked sugars. (E) Binding of PNA, which is specific for terminal Galβ1,3GalNAc glycans corresponding to non-sialyated O-linked sugars.
(TIF)

S8 Fig Substrate binding of soluble HA proteins and HA-containing vesicles. Amino acid differences between hH3 and aH3 are indicated (A). Limiting dilutions of soluble H3 proteins complexed with a strepMabClassic-HRP and rabbit-α-mouse-HRP were used in the fetuin-(B) or transferrin-(C) binding assay. Optical density at 450 nm (OD450) corresponds to binding of HA to glycoproteins. Membrane vesicles obtained after co-expression of full length hH3 and hN2 or aH3 and hN2 were analysed for their ability to bind 3’SLNLN (D) or 6’SLNLN (E) in the presence of OC using BLI similarly as described in the legend to Fig 2. Representative experiments are shown.
(TIF)

S9 Fig NA activity of recombinant viruses and soluble N2 proteins as determined by ELLA. Virus preparations were normalized based on their MUNANA activity. Fetuin- (A) or transferrin- (C) coated plates were incubated with serial 3-fold dilutions of viruses (in the absence of OC). Cleavage of SIAs from glycoproteins was monitored using ECA, which binds desialylated glycans. OD450 values reflect lectin binding. Values obtained in the presence of OC (which blocks the NA protein) were considered as background values and subtracted from the values obtained in the absence of OC. OD450 values observed at a single dilution indicated by the vertical arrow in (A) and (C) are shown in (B) and (D). The ELLAs were performed twice in duplicate. The mean values of these experiments are shown. Stars depict P values calculated using one-way ANOVA (*, P<0.05).
(TIF)

S10 Fig Example of a Nanoparticle tracking analysis (NTA) experiment. Example of a NTA experiment performed using a NanoSight NS300 instrument with a hH3aN2 virus stock is shown. The black line indicates the mean of 5 measurements, while the red bars indicate the standard deviations. Mean diameters of the particles (in nm) of the different peaks are indicated. The main peak contains particles with an average diameter of 118 nm. The different virus preparations analysed in this study displayed similar particle-size distributions.
(TIF)
