# Supplementary figures and images for: The 2nd sialic acid-binding site of influenza A virus neuraminidase is an important determinant of the hemagglutinin-neuraminidase-receptor balance
Source: PLoS Pathog. 2019 Jun 10;15(6):e1007860. doi: 10.1371/journal.ppat.1007860 (PMC6586374; doi:10.1371/journal.ppat.1007860)

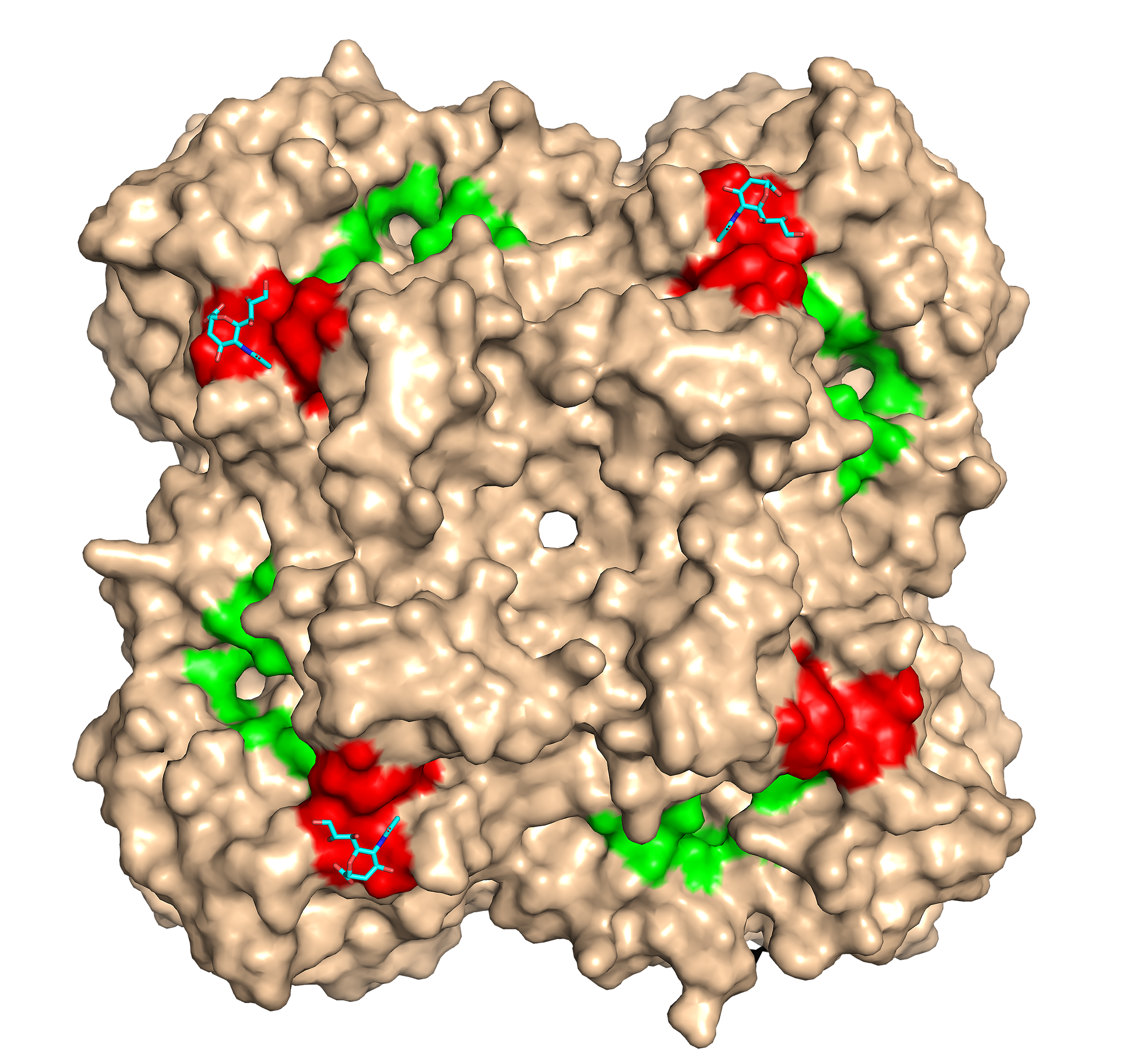

Supplement: S1 Fig — (A) Surface representation of the crystal structure of the N2 from pandemic A/RI/5 +/1957 (H2N2) in complex with Neu5Ac (PDB ID:4H53; [70]) was depicted using Pymol software. Top view is shown. The SIA-contact residues in the NA active site and the 2SBS are coloured green and red, respectively. The Neu5Ac moieties in the 2SBS sites are shown in a stick representation. (TIF) [file ppat.1007860.s001.tif]

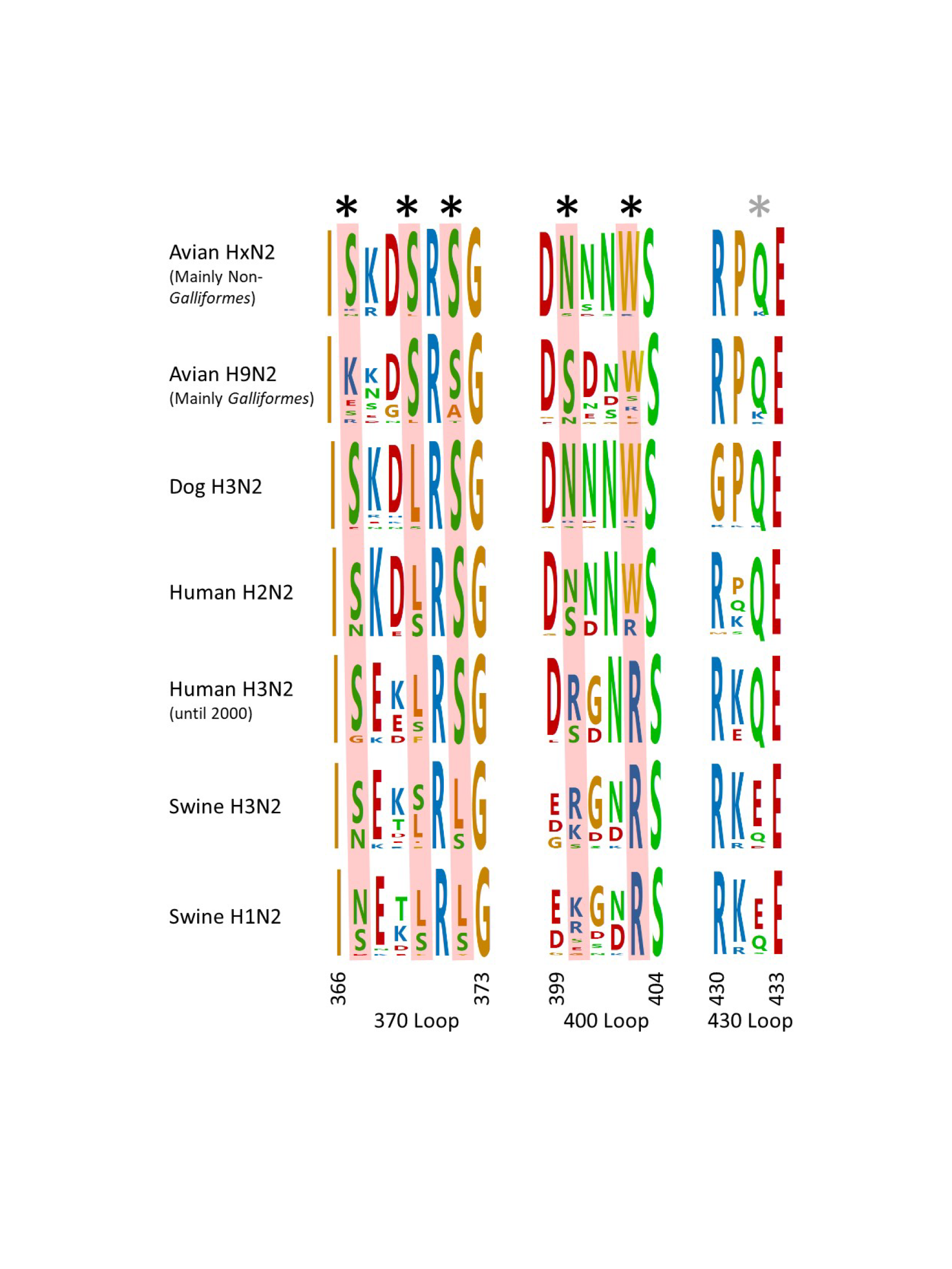

Supplement: S2 Fig — Sequence logos were generated for the three loops (370, 400 and 430 loop) that constitute the 2SBS using DNASTAR Lasergene 14 software (MegAlign Pro 14). The overall height of the stack indicates the sequence conservation at that position, while the height of symbols within the stack indicates the relative frequency of each amino acid at that position. All sequences available for avian viruses containing N2 excluding H9N2 (indicated by Avian HxN2), avian H9N2, dog H3N2, human H2N2, human H3N2 until 2000, swine H3N2 and swine H1N2 from the Influenza Research Database (https://www.fludb.org/) were used. SIA-contact residues were highly conserved in Avian HxN2, but not in H9N2 viruses. Avian H9N2 viruses were mainly (>80%) found in Galliformes species (chicken, turkey and quail), while avian HxN2 viruses were isolated mainly from non-Galliformes species (>75%). Dog H3N2 viruses generally contain a S370L mutation in the 370 loop, which is known to affect functionality of the 2SBS [28], while in addition the identity of the 430 residue deviates from those found in avian viruses. Please note that the phylogenetic analysis shown in S3 Fig indicates that human H2N2 viruses either have a mutated SIA-contact residue at position 367 or at position 370, both of which are known to disrupt the 2SBS [28]. Swine viruses containing N2, which are generally derived from human viruses [71], also contain a mutated 2SBS. SIA-contacting residues were labelled with asterisks in the sequence logo of the avian HxN2 viruses. The grey asterisk indicates an additional SIA-contact residue in the 430 loop of N9. Numbering of the start and end residues of the three loops is indicated. (TIF) [file ppat.1007860.s002.tif]

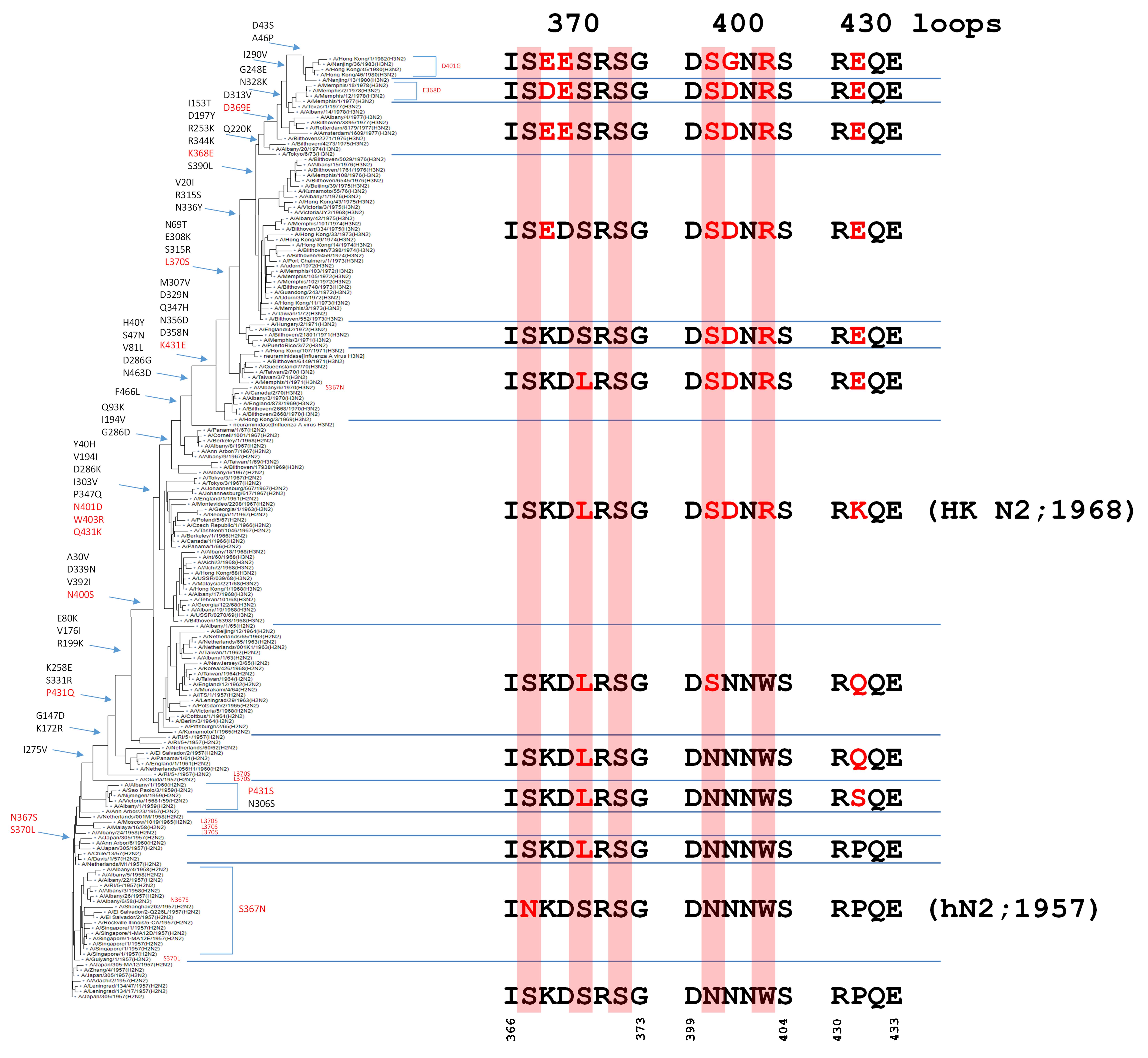

Supplement: S3 Fig — All full-length and unique N2 protein sequences of human H2N2 and H3N2 viruses between 1957–1980 were downloaded from the GenBank and GISAID databases. N2 protein trees were constructed by using the PHYLIP neighbor-joining algorithm with the mPAM distance matrix. This tree was used as a guide tree to select N2 sequences representing all main branches of the tree. The selected N2 proteins were used to construct a summary tree with topology similar to that of the guide tree. Mutations that became fixed along the trunk of the tree are indicated as well as 2SBS residues that differ between different branches. On the right site the residues of the 370, 400 and 430 loops that make up the 2SBS are shown. SIA-contact residues in the N2 protein are indicated by the red shading. Mutations in N2 relative to the avian consensus sequence are shown in red. (TIF) [file ppat.1007860.s003.tif]

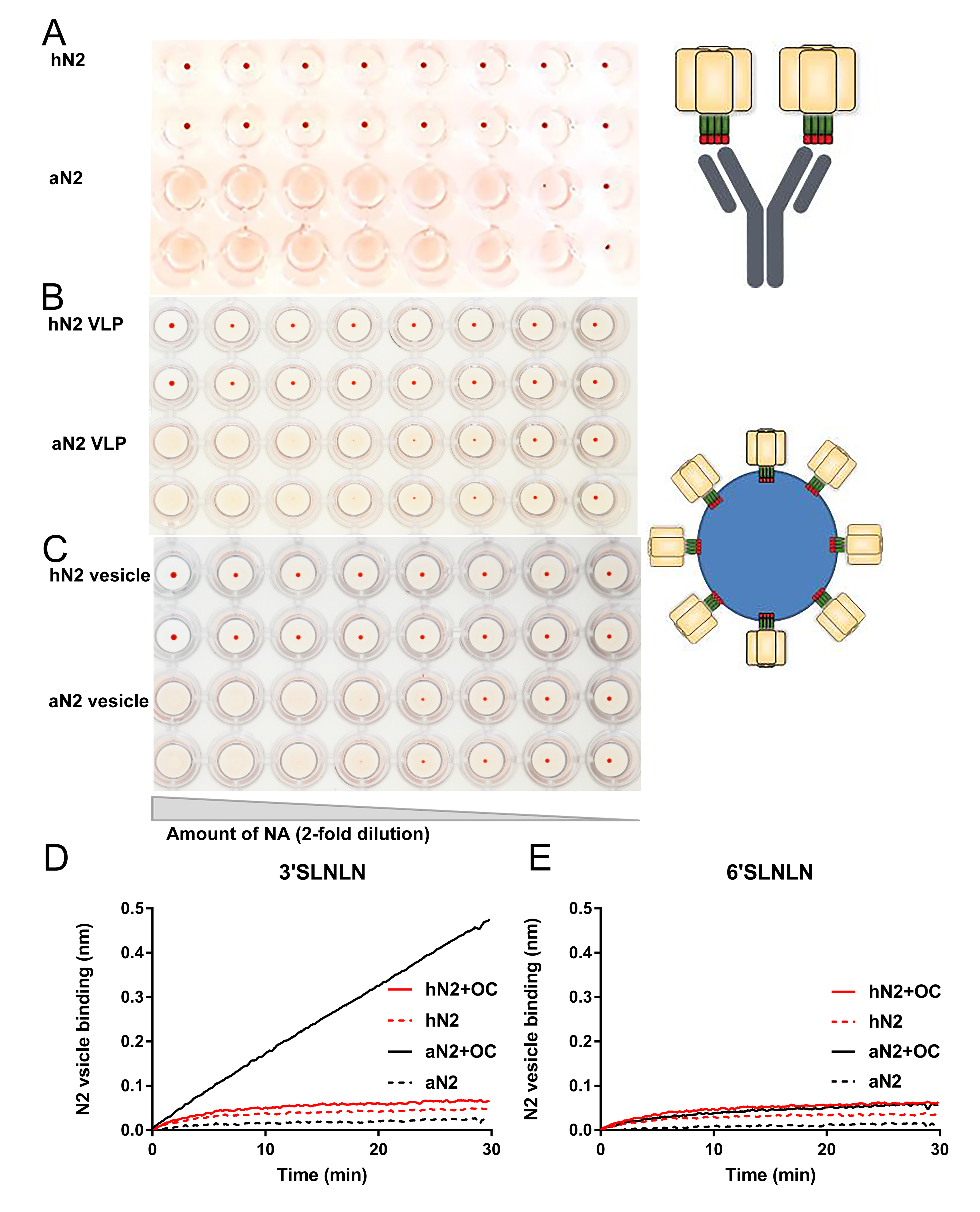

Supplement: S5 Fig — (A) Identical amounts of recombinant soluble hN2 and aN2 protein were pre-complexed with a strepMabClassic-HRP and rabbit-α-mouse-HRP prior to their incubation with erythrocytes. Serial twofold dilutions of the antibody-N2 complexes were incubated with equal volumes of 0.5% human erythrocytes at 4°C for 2 h in the presence of OC. Red dots at the bottom of the wells indicate absence of hemagglutination. (B-C) Hemagglutination using membrane vesicles (B) or VLPs (C) containing identical amounts of N2 protein. Membrane vesicles containing full length hN2 and aN2 were analysed for their ability to bind 3’SLNLN (D) or 6’SLNLN (E) in the absence or presence of OC using BLI similarly as described in the legend to Fig 2. Representative experiments (out of three performed) are shown. (TIF) [file ppat.1007860.s005.tif]

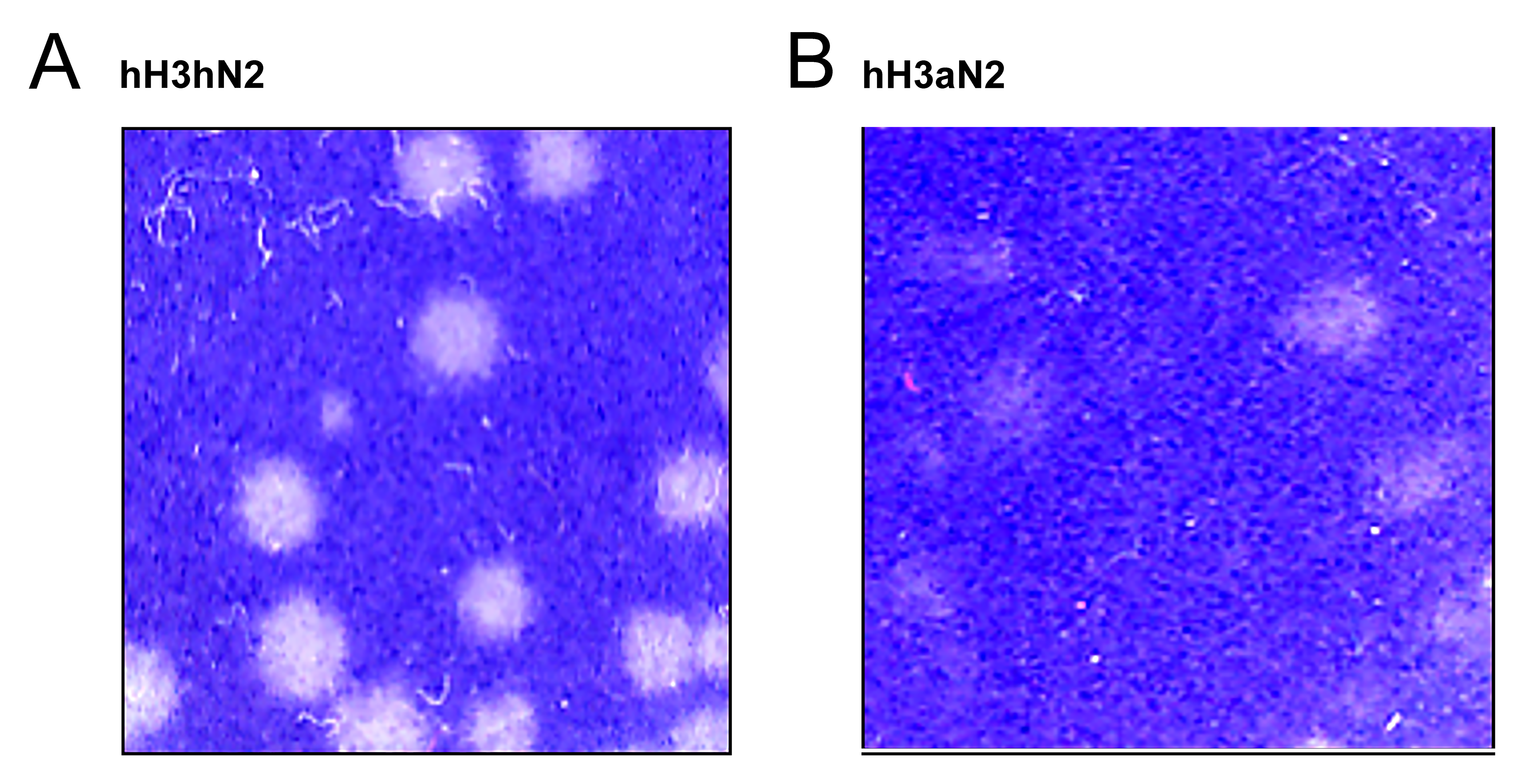

Supplement: S6 Fig — Plaque assays were performed for hH3hN2 (A) and hH3aN2 (B) viruses using Vero cells followed by crystal violet dye staining. (TIF) [file ppat.1007860.s006.tif]
